# Recognising stillbirth as a loss of life and not a baby born without life

**DOI:** 10.1136/bmjgh-2023-011815

**Published:** 2023-03-07

**Authors:** Rakhi Dandona, Carl Tollef Solberg

**Affiliations:** 1 Public Health Foundation of India, Gurugram, Haryana, India; 2 Institute for Health Metrics and Evaluation, University of Washington, Seattle, WA, USA; 3 Centre for Medical Ethics, Department of Health and Society, University of Oslo, Oslo, Norway

**Keywords:** Child health, Health policy, Maternal health, Public Health

**Summary**

- Stillbirths and their families continue to be neglected despite several calls to address preventable stillbirths.
- The dichotomy between stillbirth and neonatal death in the quantification of loss does not comply well with the societal burden of perinatal deaths or with the philosophical accounts of death’s individual harm.
- Grief is a natural emotional consequence of attachment and loss, whether the loss of a limb, country, employment, marriage or other crucial relationships. We argue that giving birth to a baby bearing no signs of life is grief unlike any other. Grieving for death must be rebalanced to include stillbirths.
- Recognising stillbirth as a loss of life and not a baby born without life is important for the global child survival initiatives to be effective in reducing preventable stillbirths.

The proposition of the *Lancet Commission on the Value of Death* is that our relationship with death and dying needs rebalancing because how people die has changed radically over recent generations as death comes later in life for many and dying is often prolonged, and has moved from a family and community setting to primarily the domain of health systems.^1^ They argue that rebalancing death and dying will depend on changes across death systems—the many interrelated social, cultural, economic, religious and political factors that determine how death, dying and bereavement are understood, experienced and managed. We support this rebalancing of death and dying and suggest a broader scope for it by the inclusion of stillbirths—babies born dead. The incident of ‘death’ (loss of one’s life) impacts the friends and family left behind in addition to the individual who loses his/her own life. We argue that this type of impact is also true for stillbirths because a stillbirth is still a birth. Despite several calls to address preventable stillbirths, the acknowledgement that these babies ‘die’ and hence are born dead, and that some of them could and should have been born alive continues to be neglected by health practitioners, policy makers and in health metrics indicators.^2 3^

The recent UNICEF-IGME report estimated nearly 2 million stillbirths globally in 2021, defined as fetal death at or after the 28th gestational week but before birth.^3^ In comparison, an estimated 2.4 million neonatal deaths occurred globally in 2019, which is the death of a newborn (live birth) between birth and the first 28 days of postpartum life.^4^ The most disability-adjusted life-years (DALYs), approximately 86 DALYs, in the Global Burden of Disease Study arise from neonatal death, most of which are early neonatal deaths that occur at birth (intrapartum complications) or within the first 6 days postpartum. Notably, many neonatal deaths result from preterm birth—that is, birth earlier than 37 weeks of gestation. Therefore, in terms of the burden of disease, a baby born alive and prematurely at the 24th gestational age who dies at birth or right after birth is registered as the worst possible tragedy with 86 DALYs. In contrast, the death of a baby in the womb at 40th week of gestation just before birth (stillbirth) is not assigned any disease burden.^5^

Today’s majority view for contemporary philosophers is that death is comparatively harmful to the individual who dies,^6 7^ and the years of life lost component of the DALY relies on such counterfactual reasoning.^8 9^ In this philosophical reasoning, death implies a loss of a future, and generally, death at a young age results in losing a more extensive future than death at an older age. If taken seriously, such a comparative account of the harm of death implies that neonatal death is considered not just death of the neonate but death of ‘a future like ours’ with all that life has to offer. That is to say, the death of a baby implies the loss of not only the baby itself but also the child and adult person that it could have been had it not died. However, the dichotomous view that birth itself constitutes the difference between a seemingly morally insignificant event (ie, stillbirth) and the worst tragedy we can think of (ie, neonatal death) neither complies well with the philosophical perspective nor with the empirical literature on the societal burden of perinatal deaths.^6^ ^10^ There is also no birth dichotomy in perinatal medicine but rather a set of overlapping pathologies that can occur both before and after birth. The built-in ethical tension of perinatal deaths is also well reflected in the etymology of ‘burden’ itself, which can mean both ‘to bear children’ and ‘that is borne’. Thus, we believe that our concept of disease burden should ideally reflect not only the harm of perinatal deaths that occur after birth but also those that occur before birth. The babies who are stillborn are real babies, and just because they died before birth does not mean they did not exist. And yet stillbirths are also overlooked in fertility indicators such as the crude birth rate which is based only on livebirths,^11^ and in vital registration systems in many countries.^12^

The *Lancet Commission* describes grief as the natural emotional consequence of attachment and loss, whether the loss of a limb, country, employment, marriage or other crucial relationships and mourning as the public face of this grief.^1^ Similarly, the devastating incomprehension of giving birth to a baby bearing no signs of life is unexplainable. There is no greater pain that a parent can feel than leaving the hospital with empty arms without the baby and coming home to a house prepared for a baby that did not make it home. However, the invisibility of stillbirths is apparent even in grief and mourning, as individual feelings of guilt or shame prevent public mourning of their loss.^10^ This lack of opportunity to publicly grieve fuels the cycle of stillbirths being considered of less consequence and without merit of grieving, contributing to their invisibility. Furthermore, bereavement refers to losing an important relationship through death and can be associated with many physical and mental health problems. The loss of a baby born dead reaches far beyond the loss of life. The psychological costs, including maternal depression and its impact on fathers, family and siblings, are profound and long-lasting.^10 13^ During the COVID-19 pandemic, the world saw people dying alone and families unable to say goodbye and being prevented from coming together in grief.^14^ This has been the case since long for many stillborn babies as they are not given proper burial or goodbyes.^13^

The birth of a dead baby impacts families, and the most impact is on the mother. She enters the hospital pregnant but leaves with a box or empty arms. With women traditionally viewed as caregivers at times of ill health and dying, it is estimated that women contribute almost 5% of the global gross domestic product through health caring.^15^ However, caregiving support is not always available to the mother of a baby born dead, who feels undervalued and unsupported having given birth to a baby born dead.^16^ If current trends continue, an additional 20 million stillbirths are estimated to occur before 2030, placing an immense burden on women, families and society.^3^ Therefore, there are reasons to argue that death’s harm to others implies that there should be no prebirth and postbirth dichotomy for either quantifying the disease burden or being able to grieve and be supported.

The world suffered an estimated 48 million stillbirths in the past two decades. The health community recognises the urgent need to prevent stillbirths, and stillbirth prevention has become an essential part of global child survival initiatives.^3^ The UN-IGME report has highlighted urgent actions to prevent an estimated 20 million more stillbirths by 2030.^3^ Importantly, this death toll could likely be higher because of the impact of COVID-19.^17^ The *Lancet Commission* emphasises that grieving must be rebalanced and calls on the society to respond to this challenge.^1^ We respectfully extend this challenge and call on society to embrace stillbirths as the death of a baby, many of whom should have been born alive, which is essential not only for the global child survival initiatives to be effective in preventing further loss of lives but also for providing support for those grieving the loss of lives of their babies.

In conclusion, real progress in stillbirth prevention can be made by simply recognising stillbirth as a loss of life and not a baby born without life. There is still a pregnancy, still a baby, still a mother, still a father—a stillbirth is still a birth. Let’s grieve for a whole life lost.

## Data Availability

No data are available.
